# Playerload™ and accelerometer-based metrics: scientific evaluation and implications for athlete monitoring

**DOI:** 10.3389/fspor.2025.1710693

**Published:** 2026-01-12

**Authors:** Craig A. Staunton, Peter Edholm, Bernardo N. Ide, Massimiliano Ditroilo, Daniel Wundersitz

**Affiliations:** 1 Swedish Winter Sports Research Centre, Department of Health Sciences, Mid Sweden University, Östersund, Sweden; 2 Department of Environmental and Bioscience, School of Business, Innovation and Sustainability, Halmstad University, Halmstad, Sweden; 3 Exercise Science, Health and Human Performance Research Group, Department of Sport Sciences, Institute of Health Sciences, Federal University of Triângulo Mineiro, Uberaba/MG, Brazil; 4 School of Public Health, Physiotherapy and Sports Science, University College Dublin, Dublin, Ireland; 5 Holsworth Biomedical Research Centre, La Trobe Rural Health School, La Trobe University, Bendigo, VIC, Australia

**Keywords:** Accel'Rate, IMU, mechanical variables, misuse, training monitoring

## Abstract

PlayerLoad™ is the most widely used accelerometer-derived metric for quantifying external demands in sport. Its normalized variant, PlayerLoad·min^−1^, is also commonly used as a marker of exercise intensity. However, recent literature has raised concerns regarding its scientific foundation, including inconsistent definitions, arbitrary units, opaque filtering methods, questionable theoretical underpinnings, and imprecise mechanical terminology. The construct validity of PlayerLoad™ remains unverified, and emerging evidence suggests weak dose–response relationships with performance outcomes. Although widely adopted in practice, these concerns warrant critical scientific scrutiny. This review critically evaluates the validity and reliability of the PlayerLoad™ metric, highlighting the need for greater transparency and theoretical rigor in wearable athlete monitoring. Furthermore, we present alternative accelerometer-derived metrics, developed from clearer biomechanical and physiological principles, which may offer more robust and interpretable measures for researchers and practitioners.

## 1 Introduction

Accelerometers, commonly integrated into wearable inertial measurement units (IMUs), have become widely used tools for quantifying athlete movement due to their ability to objectively capture three-dimensional motion at high sampling frequencies (1–5). Unlike technologies such as Global Positioning Systems (GPS), accelerometers are suitable for both indoor and outdoor environments. Additionally, their compact size, lightweight design, and relatively low cost also facilitate seamless integration into athlete clothing and equipment, enhancing their practicality for sports performance monitoring (6).

Accelerometers also address several limitations associated with other athlete monitoring modalities. For instance, video tracking and GNSS/GPS technologies primarily capture horizontal displacement and lack sensitivity to vertical or rotational movements. Physiological measures such as heart rate (HR) exhibit inherent delays in responding to rapid changes in exercise intensity (7), are limited in their ability to assess anaerobic effort, and are heavily influenced by external and internal factors such as ambient temperature and hydration status (8). Subjective measures like rating of perceived exertion (RPE) are susceptible to individual mood, personality traits, and recall bias (9, 10), which can compromise their reliability.

One of the most widely used accelerometer-derived metrics for quantifying athlete movement is PlayerLoad™ (PlayerLoad), a proprietary algorithm developed by Catapult Sports. Since its introduction, PlayerLoad has become the most frequently reported accelerometry-based metric in sports science (11–14). PlayerLoad is reported in arbitrary units and is purportedly calculated as the cumulative magnitude of the rate of change of acceleration (2, 3), a quantity commonly referred to in mechanics as “jerk” (15). However, the exact calculation method remains proprietary, with limited methodological transparency provided by the manufacturer. A time-normalized variant, PlayerLoad·min^−1^, is often used as an indicator of external intensity in elite sports such as Australian football (13, 14), netball (4, 14, 16) and soccer (17–19). This normalization aims to account for differences in session duration, potentially allowing for comparisons across drills or time periods within the same athlete.

Some studies have reported excellent test–retest reliability for PlayerLoad, suggesting potential utility in quantifying athlete movement demands (1, 2). Additionally, moderate to strong correlations have been observed between PlayerLoad and commonly used measures of internal demands, such as HR and RPE, which some authors interpret as evidence of convergent validity (5, 12, 18, 20). However, this interpretation may be circular: the validity of PlayerLoad is inferred from its association with internal measures that themselves have notable limitations (21). For example, HR and RPE can increase due to heat stress, dehydration, or psychological factors even when the athlete's external mechanical demand and therefore PlayerLoad, remains relatively unchanged (22–24). Conversely, PlayerLoad can rise due to high-frequency, low-metabolic activities such as repeated changes of direction, technical footwork, or short accelerations that have minimal impact on HR or RPE (25, 26). In these situations, correlations between PlayerLoad and these measures may simply reflect occasions when both variables trend upward for different reasons, rather than indicating that PlayerLoad is a valid measure of overall physical demand. As a result, such associations may reflect shared limitations in the measures rather than providing robust evidence of construct validity.

Despite its widespread use, recent literature has highlighted multiple limitations of PlayerLoad (27–31). These include inconsistent definitions and calculation methods (28), lack of theoretical justification for its derivation (31), limited transparency in manufacturer-applied data filtering procedures [“opacity”; (30)], reliance on arbitrary units for quantification (27, 29), and imprecise or misleading use of mechanical terminology (29). Although some studies report evidence of convergent or concurrent validity (1, 30, 32), the construct validity of PlayerLoad, that is, its ability to accurately represent the external demands of sport-specific activities, has not been demonstrated. An increase in PlayerLoad with greater movement does not inherently justify its use as a proxy for external intensity or training volume. The time-normalized variant, PlayerLoad·min^−1^, is often used to account for session duration and infer exercise intensity. However, its interpretation is complicated by the same underlying issues as PlayerLoad itself, including arbitrary units and lack of biomechanical specificity. Moreover, its use across athletes is problematic due to individual differences in movement mechanics and sensor placement, limiting its utility for between-athlete comparisons. Moreover, evidence for meaningful dose–response relationships between PlayerLoad and performance adaptations remains weak (33, 34). Taken together, these concerns highlight the need for more rigorous scrutiny and theoretical clarity in the ongoing use of PlayerLoad within athlete monitoring frameworks.

Accordingly, this review provides a critical evaluation of the PlayerLoad metric within sports science. By integrating evidence from biomechanics, signal processing, and applied sport science, this review raises awareness of the methodological and conceptual challenges associated with proprietary accelerometry metrics. It also introduces alternative accelerometer-derived metrics that are developed on clearer biomechanical and physiological principles, offering researchers and practitioners tools that are more interpretable and potentially more valid for athlete monitoring.

In doing so, this review provides a consolidated framework that identifies both the strengths and weaknesses of current approaches, and outlines actionable directions for future research, including empirical validation, construct clarification, and integration of accelerometry with complementary monitoring indicators. This perspective aims to guide both applied practitioners and the scientific community toward more robust and meaningful use of athlete monitoring tools.

To support clarity and consistency in the sections that follow, key accelerometry-related terms used throughout this review are defined in Table 1.

**Table 1:** Key mechanical and signal-processing terms used in this review.

Term	Definition
Acceleration	The rate of change of velocity over time; derived from the second derivative of position. In IMUs, measured along three orthogonal axes. SI unit: m·s^−2^
Resultant acceleration	The magnitude of the 3D acceleration vector, calculated as √(*ax*^2^ + *ay*^2^ + *az*^2^).
Net acceleration	Acceleration magnitude after subtracting static gravitational acceleration; intended to approximate segment or whole-body dynamic acceleration.
Jerk	The rate of change of acceleration over time (third derivative of position). Reflects rapid transitions in movement or impacts. SI unit: m·s^−3^
Force	A mechanical quantity defined as mass × acceleration (*F* = ma). Not directly measured by IMUs but sometimes inferred or estimated. SI unit: N
Impulse	The integral of force over time; represents the change in momentum produced during a movement. SI unit: N·s
Vector	A quantity with both magnitude and direction; IMU axes provide vector components (*x*, *y*, *z*).
PlayerLoad™	A proprietary metric defined as the square-root of the sum of squared instantaneous changes in acceleration across three axes, divided by 100. Intended to represent “overall load,” though its mechanical interpretation is unclear.
Clipping/saturation	Occurs when sensor acceleration exceeds the sensor's dynamic range (e.g., ±16 g), causing the recorded signal to flatten at the maximum values, distorting true motion.
Dynamic range	The maximum and minimum measurable acceleration values of a sensor. Determines whether high-intensity movements can be accurately captured.
Sampling frequency	The number of data samples recorded per second (Hz). Determines the highest resolvable motion frequency (Nyquist limit).
High-pass filter	Removes low-frequency components of a signal (e.g., drift, orientation changes), preserving high-frequency content such as impacts.
Low-pass filter	Removes high-frequency noise and retains slower, smoother components of the signal (“movement-level” dynamics).
Band-pass filter	Retains a specific range of frequencies while removing both lower and higher frequencies; used to isolate certain types of movement signatures.

### 1.1 Accelerometers

A comprehensive understanding of accelerometry is essential for accurately interpreting accelerometer-derived metrics, such as PlayerLoad, within the context of athlete monitoring. This requires clarity on what accelerometers are, what they measure, and how their data are processed.

Accelerometers are sensitive electromechanical sensors that measure the magnitude and direction of acceleration. They function by converting mechanical movement into digital signals representing acceleration along one or more axes (35). Acceleration is a vector quantity defined as the rate of change of velocity with respect to time (Equation 1) and is typically expressed in SI units as meters per second squared (m·s^−2^). Accordingly, when an individual wearing an accelerometer changes velocity, the device captures the resulting acceleration of body segment to which it is attached.

Average (*av*) and instantaneous (*x*) acceleration.

$$\begin{array}{rl} & {\vec{a}}_{av}=\frac{\Delta v}{\Delta t}\\ & {\vec{a}}_{x}=\underset{\Delta_{t}\to 0}{\lim}\frac{\Delta_{vx}}{\Delta_{t}} \end{array}$$ (1)

*a* = *acceleration*, *v* = *velocity*, *t* = *time*, Δ=change.

It is important to recognize that accelerometers measure accelerations from multiple sources, including:

1. **Body segment movement**—assuming the accelerometer is rigidly coupled to the body segment, this reflects actual movement-related acceleration.
2. **Gravitational acceleration**—approximately 9.81 m·s^−2^ on Earth (equivalent to 1 g), which is always present and must be accounted for.
3. **Low-frequency noise or DC offset**—such as electrical noise within the device, which can distort baseline readings.
4. **High-frequency noise**—including sensor vibrations or perturbations caused by external factors like movement within a harness or clothing.

In sports applications, the primary interest lies in capturing acceleration generated by body movement, while minimizing the influence of extraneous sources that introduce noise into the signal. Signal processing techniques, such as filtering (discussed further in Section 2.1), are commonly employed to attenuate noise and improve data accuracy. Historically, exercise science research has utilized uniaxial (single axis) accelerometers to quantify physical activity across various populations and movement types (36–39). These devices have been applied to estimate metrics such as energy expenditure and step counts during ambulatory activities like walking and low-speed jogging (40–45). However, due to the complex, multidirectional nature of movements in team sports, capturing acceleration across multiple planes is essential for accurate monitoring.

**Figure 1:**
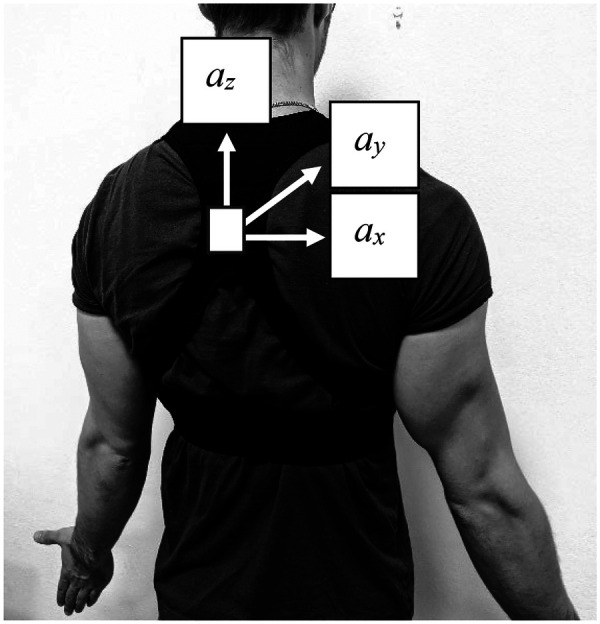
Typical location of wearable devices used in team sports, commonly integrated with a triaxial accelerometer. *a_x_*, mediolateral acceleration; *a_y_*, anteroposterior acceleration; *a_z_*, vertical acceleration.

Consequently, triaxial accelerometers, which measure acceleration along three orthogonal axes: anteroposterior, mediolateral, and vertical (see Figure 1), have become the standard in sports monitoring. Compared to uniaxial devices, triaxial accelerometers have demonstrated improved accuracy in estimating energy expenditure (46) and show stronger correlations with oxygen consumption (47, 48). These devices are now widely adopted for quantifying external demands in elite team sports including Australian football (2, 13), netball (4), soccer (12, 18) and basketball (5, 49).

### 1.2 Signal processing

Signal processing refers to the analysis and modification of raw accelerometer data to enhance signal quality and emphasize components relevant to human movement (50). A fundamental aspect of this process is filtering, which is used to reduce noise and improve data interpretability. However, before filtering can be effectively applied, two critical factors must be considered during the acquisition: *sampling frequency* and *device dynamic range*.

*Sampling frequency* refers to the number of acceleration samples recorded per second (Hz). Accurate capture of human movement requires adherence to the Nyquist–Shannon Sampling Theorem (51, 52), which states that the sampling frequency must exceed twice the highest frequency present in the signal to avoid aliasing (35). Aliasing occurs when higher-frequency components are misrepresented as lower-frequency signals due to insufficient sampling, causing rapid or high-intensity movements to appear slower or less intense than they are in reality (53).

Frequency-domain analyses (e.g., FFT) indicate that most human movement energy is concentrated between ∼0.6–5 Hz (53–55), although specific actions such as rapid arm movements can reach ∼25 Hz (53), and even higher frequencies have been documented during intense sporting tasks (56). Accordingly, while a minimum of 50 Hz is required to capture fast movement components, sampling rates of 100 Hz or higher are commonly used in team sports to ensure accurate representation of rapid changes in direction and high-intensity actions (4, 5, 13, 14, 16, 49).

Another key parameter is the *dynamic range* of the accelerometer, which refers to the maximum and minimum acceleration values that the device can measure. This is typically expressed in units of gravitational acceleration (g). If the dynamic range is insufficient, the sensor may clip acceleration peaks that exceed its limits, resulting in data loss. For example, if an accelerometer has a dynamic range of ±16 g but it experiences a 20 g acceleration, values above 16 g will be clipped and not recorded. Peak accelerations measured at the ankle during running can reach up to 12 g (57, 58) and vertical impacts from jump landings can exceed 20 g (59). However, peak accelerations recorded using trunk-mounted accelerometers are generally lower, typically not exceeding 5 g during running (60). Despite this, some studies have reported trunk-mounted peak accelerations greater than 10 g during high-impact activities such as tackling and bumping (61). Therefore, when using a trunk-mounted accelerometers in sport settings, a dynamic range of at least ±12 g is recommended to ensure all relevant movements are captured. For example, Catapult Sports' Vector^TM^ devices feature a dynamic range of ±16 g.

After data acquisition, raw acceleration signals must be processed to remove signal noise and emphasize components relevant to human movement (61–64). A key aspect of this process is filtering, which reduces artefacts and signal components unrelated to movement. For instance, low-frequency signals such as gravitational acceleration and electrical noise can be attenuated using a high-pass filter. Some researchers and practitioners attempt to simplify this problem by subtracting 1 g from the acceleration magnitude. While this may seem intuitive, it is a crude approach that assumes the device remains perfectly aligned with gravity and that gravitational acceleration contributes equally to the resultant signal at all times. In reality, sensor orientation changes continuously during movement, causing the gravitational vector to project differently across each axis. As a result, naïve subtraction of 1 g fails to reliably isolate inertial acceleration and can introduce substantial error. By contrast, high-pass filtering offers a more robust solution. It removes the near-constant gravitational component and very low-frequency drift while preserving the higher-frequency signals associated with dynamic human movement.

High-frequency noise arising from device vibrations or sensor perturbations can be mitigated using a “low-pass filter”, which attenuates signals above a specified frequency threshold. Conversely, a “band-pass filter” combines both low- and high-pass filtering, allowing only signals within a defined frequency range to pass through while removing frequencies outside this window. Selecting appropriate filter cut-offs is critical to preserving meaningful data while minimizing artefacts. If the high-frequency (low pass) cut-off is set too high, residual noise unrelated to physical movement (e.g., mechanical vibrations or electrical artefacts) may remain in the signal. In contrast, setting the cut-off too low risks attenuating or eliminating relevant movement data. In team sports applications, research suggests optimal high-frequency cut-offs between 10 Hz and 16 Hz for typical movement patterns (62, 64) and up to 20 Hz for capturing high-impact events such as physical collisions (61). For low-frequency (high-pass) filtering, which aims to remove gravitational components and low-frequency device noise, optimal cut-off frequencies have been reported between 0.08 Hz and 0.23 Hz (65, 66).

Filtering choices directly influence the accelerometer output and, consequently, affect calculations of athlete activity demands (67). However, a significant challenge in the field is the lack of transparency in filtering methodologies, particularly among commercial manufacturers. For example, the exact filtering parameters used in the calculation of PlayerLoad remain undisclosed due to proprietary constraints (68). A recent systematic review highlighted both the wide variety of filters employed and a concerning lack of reporting of signal processing methods in the literature. Only 13% of published studies provided details on the filters used to process athlete movement data (67). While manufacturers may understandably protect proprietary algorithms for competitive reasons, this opacity raises ethical and potential legal concerns (69, 70). Further, it undermines scientific reproducibility, a cornerstone of research integrity (71), and complicates cross-study comparisons of athlete demands. Without detailed signal processing information, it is impossible to determine whether observed differences in external demands reflect true variations in activity intensity or are merely artefacts of differing filtering approaches (67, 72).

Therefore, researchers and practitioners must remain cognisant of the limitations associated with proprietary metrics. To enhance the consistency and comparability of accelerometry-based research, the adoption of standardized and transparent data processing methods is strongly recommended. While commercial metrics are widely used due to their user-friendliness, it is essential that researchers report key technical details in publications, including: device brand and model; software and firmware versions, and all available information on signal processing methods (73). In summary, filtering improves accelerometer signal quality by reducing noise and emphasizing signals relevant to human movement. Based on current evidence, we recommend the following guidelines for effective signal processing:

1. Use a high-frequency (low-pass) filter cut-off between 10 Hz and 20 Hz, depending on the activity type, to reduce high-frequency noise (61, 62, 64).
2. Apply a low-frequency (high-pass) filter cut-off around 0.1 Hz to remove low-frequency noise such as gravitational acceleration (65, 66).
3. Employ accelerometers with a dynamic range of at least ±12 g to accurately capture high-intensity movements.
4. Use sampling frequencies of at least 100 Hz to satisfy the Nyquist-Shannon criterion and ensure signal fidelity (51, 52).

### 1.3 Attachment location

Accelerometers have been attached to a variety of anatomical locations in human movement research, including the chest (74), waist (40), ankles (75), lower back (49), wrist (76), upper back (3), and head (77). Each placement captures slightly different biomechanical signals due to variations in local segmental movement, soft tissue artefact, and proximity to the body's centre of mass (35). The influence of sensor location on output has been empirically demonstrated. For example, Altini et al. (78) compared accelerometer-derived signals from five anatomical sites (chest, lower back, wrist, and both hips) during identical movements and found that signal output varied by up to 12% depending on placement. In a sports-specific context, Barrett et al. (1), reported that PlayerLoad values obtained from a unit mounted on the upper back were approximately 16% lower than those recorded at a location closer to the centre of mass during treadmill running.

The centre of mass is generally regarded as the most appropriate location for measuring overall dynamic body acceleration, as it best reflects whole-body movement patterns (79). However, in applied sports settings, the upper back, specifically the region between the scapulae, is by far the most common attachment site. This standardisation is largely driven by practical considerations: device manufacturers typically provide form-fitting harnesses (e.g., compression vests and sports bras) designed to securely house the device at this location. Furthermore, for systems incorporating GPS or GNSS functionality, upper-back placement optimizes signal acquisition by improving line-of-sight with satellites (1). Safety is another contributing factor, positioning the device higher on the torso reduces the risk of injury during physical collisions, both for the wearer and for other players.

While upper-back placement is practical and widely adopted, it is important for practitioners and researchers to recognize its limitations. Signals recorded from this location reflect local segmental acceleration of the upper thorax, rather than total body acceleration. Compared to placement at the centre of mass, upper-back positioning tends to underestimate accelerations by approximately 10%–20%, depending on the activity type and movement intensity (1, 78). This attenuation must be carefully considered when interpreting accelerometer-derived metrics, particularly when comparing results across studies or using such metrics to inform athlete monitoring, management, or performance decisions.

## 2 PlayerLoadTM

With the foundational understanding of accelerometry and signal processing established, we now turn our attention to PlayerLoad, one of the most widely used metrics derived from triaxial accelerometer data in sports science. PlayerLoad was developed by Catapult Sports™, in collaboration with the Australian Institute of Sport, originally to quantify training and match demands in rugby union (see: https://support.catapultsports.com/hc/en-us/articles/360000510795-What-is-Player-Load) (80). The metric has since been adopted across a wide range of sports and settings.

In the scientific literature, PlayerLoad was first described as, “*the rate of change in acceleration in three planes of body movement: up/down (z), side/side (y) and forward/backward (x)… (and) multiplied by a scaling factor of 0.01*” (49). Later, Boyd et al. (2) formalized the calculation using a modified vector magnitude algorithm. This approach quantifies the instantaneous rate of change in acceleration across all three axes and accumulates this over time to provide an index of the total movement demands. This algorithm is presented in (Equation 2):

Accumulated PlayerLoad^TM^ algorithm (developed by Catapult Sports^TM^, Melbourne, Australia).

$$\begin{array}{r} \mathrm{PlayerLoa}d^{\mathrm{TM}}=\\ \sum_{t=0}^{t=n}\sqrt{{\left({a_{x}}_{t=i+1}-{a_{x}}_{t=i}\right)}^{2}+{\left({a_{y}}_{t=i+1}-{a_{y}}_{t=i}\right)}^{2}+{\left({a_{z}}_{t=i+1}-{a_{z}}_{t=i}\right)}^{2}} \end{array}$$ (2)

*a_x_* = *mediolateral acceleration*; *a_y_* = *anteroposterior acceleration*; *a_z_* = *vertical acceleration*.

While PlayerLoad offers a practical, integrated snapshot of movement intensity and volume, it is important to recognize that the metric is sensitive to factors such as sensor location, sampling frequency, and the filtering methods applied to raw data, all of which influence final values. As discussed previously, this sensitivity limits the ability to make direct comparisons between studies or devices unless key variables such as sensor placement, signal processing parameters, and algorithm definition are carefully controlled or clearly defined.

### 2.1 Validity and reliability

Numerous studies have evaluated the validity and reliability of the PlayerLoad metric. In this context, validity refers to the accuracy with which an instrument measures the intended construct (81), while reliability refers to the consistency of a measure across time, raters, settings, and items (82). Reliability of PlayerLoad is consistently reported as excellent, with coefficients of variation typically below 2% in both laboratory and field settings (1, 2). However, evidence supporting validity remains limited and problematic.

Validity itself is hierarchical, with construct validity encompassing face, content, and criterion validity, while criterion validity includes discriminative, concurrent, and predictive forms, and concurrent validity encompasses convergent and discriminant evidence (83, 84). PlayerLoad validity studies have primarily attempted to establish construct validity (i.e., whether the metric behaves as theoretically expected) by correlating PlayerLoad with constructs such as Edwards' TRIMP or session ratings of perceived exertion (s-RPE) (5, 12, 18, 20). Although moderate to strong correlations are often observed, these approaches are limited by the questionable validity of TRIMP and s-RPE themselves, creating a circular reasoning problem (21).

According to Bagner et al. (82), construct validity is informed by face validity, content validity, and, critically, criterion validity. As such, construct validity should ideally be established through comparisons with outcomes that may function as criterion measures, such as mechanical work, movement intensity, or functional adaptation. To date, no studies have rigorously evaluated these relationships with PlayerLoad. Strong correlations with s-RPE or TRIMP are unsurprising, given that PlayerLoad inherently reflects movement volume (1, 2) higher scores accompany longer or more intense bouts of locomotion, which also increase s-RPE and TRIMP scores, primarily due to increases in exercise duration (85, 86). Yet these associations provide only limited evidence that PlayerLoad meaningfully quantifies external movement demands or broader biomechanical outputs.

Content validity is similarly uncertain. Although PlayerLoad is marketed as a measure of external mechanical load, few studies have examined whether the accelerations it captures adequately represent the mechanical demands across diverse movements or training modalities. Its emphasis on locomotor accelerations suggests that PlayerLoad may indicate movement volume during running, but it may fail to capture strength-based tasks, upper-body movements, or multidirectional actions, indicating substantial context dependence. Criterion validity remains largely untested as well, with few studies comparing PlayerLoad against direct mechanical or physiological benchmarks such as force platforms, indirect calorimetry, or whole-body energy expenditure (e.g., doubly labelled water), leaving its relationship to actual mechanical or energetic load unclear.

In sum, while PlayerLoad demonstrates excellent reliability, its validity remains insufficiently established. Evidence for construct validity relies largely on correlations with measures that themselves have questionable validity, leading to circular reasoning. Evidence supporting content and criterion validity is sparse, and the responsiveness of PlayerLoad to meaningful changes over time remains largely unexplored. Despite its widespread use in research and applied settings, the interpretive boundaries of PlayerLoad remain poorly defined, underscoring the need for independent, high-quality research that evaluates PlayerLoad against theoretically meaningful and mechanically precise outcomes.

### 2.2 Issues

Beyond concerns regarding the validity of PlayerLoad, several issues have been identified relating to inconsistencies in its definition, calculation, and interpretation. Bredt et al. (28) were among the first to highlight discrepancies in the Cartesian formula used to calculate PlayerLoad across published studies. These inconsistencies extended not only to the mathematical expression of the formula but also to its textual descriptions. When applied to a common dataset, these discrepancies resulted in substantial variability in PlayerLoad outputs, as shown in their comparative analysis [see Table 1 in Bredt et al. (19)]. This lack of standardisation significantly compromises the interpretability, reproducibility, and comparability of PlayerLoad values across studies.

Further compounding this issue is the observation that PlayerLoad values generated by manufacturer software are consistently around 15 percent lower than those calculated manually using the standard Cartesian formula [Equation 2; (30)]. This discrepancy suggests that proprietary software applies undisclosed data processing or filtering, which poses serious challenges for research transparency and replicability. Without access to the underlying signal processing procedures, individuals who use PlayerLoad, such as sport scientists, coaches, and researchers, are unable to meaningfully interpret results, replicate findings, or perform valid comparisons across platforms or devices.

Large inter-individual variability in PlayerLoad has also been documented. For example, when team-sport athletes performed the same incremental running protocol to exhaustion, PlayerLoad values differed substantially between individuals (1). This finding suggests that PlayerLoad may lack the sensitivity or specificity needed to consistently capture external demands across athletes. It highlights the importance of individualized interpretation when using the metric.

Critiques have also been made regarding the terminology and units associated with PlayerLoad. As previously highlighted (18), the PlayerLoad formula is mathematically equivalent to the calculation of jerk, which is defined in biomechanics as the rate of change of acceleration (14). Based on this equivalence, PlayerLoad should theoretically be reported in SI units of metres per second cubed (m·s^−3^). However, PlayerLoad is typically presented in arbitrary units, which further limits its scientific rigour and interpretability (18).

**Figure 2:**
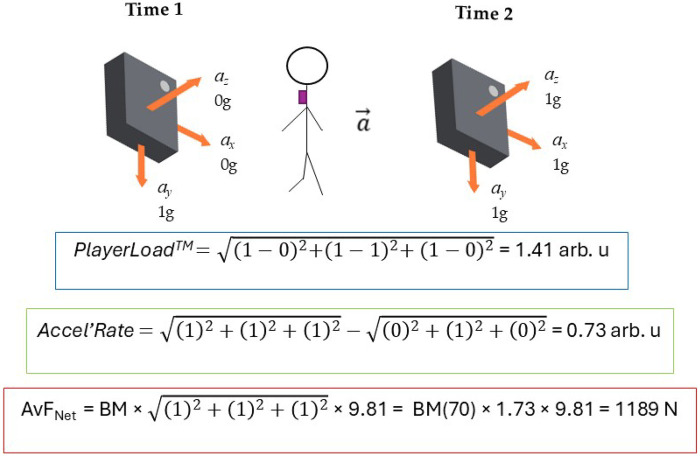
Illustration of how three accelerometer-derived metrics—playerLoad, Accel’Rate, and average Net force (AvF_Net_)—are computed from the same triaxial acceleration data. PlayerLoad and Accel’Rate both quantify the change in acceleration over time (requiring two time points), while AvF_Net_ depends solely on the resultant acceleration at a single time point (here with body mass = 70 kg). Although the same raw data are used, each metric yields very different values, highlighting their conceptual and practical differences. (illustrative values shown. In practice, AvF_Net_ should be calculated from filtered, gravity-corrected acceleration signals, e.g., dual-pass Butterworth filter, 0.1–15 Hz; Body Load and Dynamic Stress Load are not shown because their proprietary algorithms make replication complex).

Additionally, Hollville et al. (31) identified a fundamental issue in the computational formula that underpins PlayerLoad. Natural changes in device orientation during human movement can cause fictitious increases in PlayerLoad. This occurs because the metric calculates the rate of change of acceleration along each axis independently, without accounting for the rotation of the sensor as a vector. Consequently, shifts in sensor orientation, such as those caused by torso rotation or changes in body segment alignment, can produce false-positive increases in PlayerLoad. These increases arise from the redistribution of gravitational and inertial forces across axes, rather than from actual physical effort or movement. This undermines the accuracy of the metric, as illustrated in Figure 2.

Finally, Freitas et al. (87) demonstrated that PlayerLoad, because it is based on a derivative computation, is highly sensitive to noise. Moreover, the sample rate is not accounted for in the computational formula. This means that identical activity measured using two devices placed at the same location but operating at a different sampling rates will produce PlayerLoad values that cannot be meaningfully compared.

Although these issues are largely methodological, they also have practical implications for applied settings. Because PlayerLoad primarily reflects accumulated movement volume, practitioners may mistakenly interpret high values as evidence of greater mechanical stress, leading to overly conservative or overly aggressive adjustments in training. Cross-athlete comparisons can also be misleading, as athletes with different movement strategies (e.g., stiffer vs. more compliant running gait) may generate different PlayerLoad values despite performing identical work. In return-to-play contexts, reliance on PlayerLoad may give the false impression that an athlete has “matched” prior load benchmarks even when the underlying intensity and mechanical exposure differ markedly. Similarly, longitudinal trends can appear stable even when meaningful changes occur in movement quality or energetic cost. These examples highlight that misinterpretation of PlayerLoad may influence programming decisions, dose–response modelling, and athlete management.

## 3 Alternative accelerometer metrics

Given the limitations of PlayerLoad, several alternative metrics derived from accelerometer data have been proposed in recent literature (Table 2). These alternatives aim to improve both the validity and interpretability of movement-based data, offering more robust approaches to quantifying external demands.

**Table 2:** Summary of selected accelerometer-derived metrics used to quantify external demands in team-sport contexts.

Metric type	Based on	Key advantage	Limitations
PlayerLoad	Derivative of acceleration (jerk)	Commercially available	Amplifies noise; sensitive to sampling rate and orientation changes; inconsistent definitions
Accel’Rate	Derivative of acceleration (jerk)	Improves computation of PlayerLoad; validated	Not commercially available; derivative-based → sensitive to noise
Body load	Resultant acceleration magnitude	Lower noise, independent of acquisition rates	Less common commercially; arbitrary units and cut-offs
Dynamic stress load	Resultant acceleration magnitude	Lower noise, independent of acquisition rates	Proprietary algorithm; arbitrary units; complex computation
*F*_Net_	Resultant acceleration × body mass	Physically grounded; output in SI units (N); validated against VO_2_ and running speed; transparent signal processing	Sensitive to athlete body mass; requires COM placement for accuracy

PlayerLoad and Accel’Rate are derivative-based metrics sensitive to sampling rate and sensor orientation. Body Load and Dynamic Stress Load report in arbitrary units, and filtering or weighting methods are proprietary. *F*_Net_ outputs in SI units (*N* for net force; N·s for accumulated impulse) and requires placement at the athlete's centre of mass (COM) for accurate measurement. All accelerometer-derived metrics depend on appropriate signal processing (filtering, sampling frequency) to minimize noise and gravitational artifacts. SI, international system of units; COM, centre of mass.

One such metric is Accel'Rate, introduced by Hollville et al. (21), which addresses key computational limitations inherent in the PlayerLoad algorithm. Unlike PlayerLoad, which independently sums the rate of change in acceleration along each Cartesian axis, Accel'Rate calculates the rate of change of the resultant acceleration vector and integrates this value over time (see Equation 3). This vector-based approach mitigates the fictitious accumulation of acceleration caused by changes in device orientation, particularly during rotational movements. However, it does not eliminate this issue entirely.

Accumulated Accel'Rate algorithm [proposed by Hollville et al. (31)].

$$\begin{array}{r} {\mathrm{Accel}}^{\prime }\mathrm{Rate}=\\ \sum_{t=0}^{t=n}\sqrt{{\left(a_{xi}\right)}^{2}+{\left(a_{yi}\right)}^{2}+{\left(a_{zi}\right)}^{2}}-\sqrt{{\left(a_{xi-1}\right)}^{2}+{\left(a_{yi-1}\right)}^{2}+{\left(a_{zi-1}\right)}^{2}} \end{array}$$ (3)

*a_x_* = *mediolateral acceleration*; *a_y_* = *anteroposterior acceleration*; *a_z_* = *vertical acceleration*.

To evaluate the validity of Accel'Rate, Hollville et al. (21) concurrently measured triaxial accelerations and ground reaction forces using in-series force plates during running-based locomotor tasks representative of team sports. Their findings demonstrated that Accel'Rate exhibited greater concurrent validity for estimating movement-related forces than PlayerLoad, offering a more robust alternative for assessing external demands. Despite its improved validity, Accel'Rate still presents notable methodological limitations. As a derivative-based metric, it is highly sensitive to noise. Furthermore, like PlayerLoad, it disregards the sampling rate. As a result, identical activity measured by two devices placed at the same location but operating at a different sampling rates will yield incomparable results (87).

To minimize noise amplification, alternative metrics often utilize the resultant or vector magnitude of acceleration. Unlike derivative-based metrics, these approaches must account for the gravitational acceleration component, discussed earlier, which is approximately 9.81 m/s or 1 g.

One such metric based on acceleration magnitude is Body Load, developed by the manufacturer GPSports (88). Body Load subtracts 1 g from the resultant acceleration prior to calculation, includes both linear and nonlinear components, and is scaled by both the accelerometer's sampling frequency as well as an unspecified manufacturer-defined “exercise factor”. The metric is summed across all time points where the resultant acceleration exceeds 1.25 g, and is reported in arbitrary units (89). Body Load can be a valuable metric because it is independent of acquisition rates and its strong resistant to noise (87). However, it remains reported in arbitrary units, and as discussed in Section 2.1, the removal of the gravitational acceleration component by simply subtracting 1 g from the resultant acceleration is a crude approach. This method neglects the influence of sensor orientation and fails to account for low-frequency body movements that overlap with the gravitational component.

Another metric reported in the literature is Dynamic Stress Load (90), a proprietary measure developed by the manufacturer Stats Sports. It is derived from accelerometer-detected impacts, identified as peaks exceeding a threshold (e.g., >2 g within 0.1 s). Each impact is weighted using a convex, approximately cubic function, reflecting the principle that larger impacts (e.g., 4 g) impose disproportionately greater mechanical stress than smaller ones (e.g., 2 g). The weighted impacts are then summed and scaled to produce a cumulative measure of mechanical stress, reported in arbitrary units. Like Body Load, the Dynamic Stress Load offers value due to its independence from sample rate and its insensitivity to noise (87). However, as a proprietary metric, it has never been precisely defined, and attempts to replicate it have required complex computations (87).

Another promising alternative is accelerometry-derived net force (*F*_Net_), which has demonstrated validity in quantifying movement patterns and shows stronger associations with over-ground running speed than PlayerLoad (27). *F*_Net_ is calculated by applying a 4th-order Butterworth band-pass filter (0.1–15 Hz) to the three orthogonal acceleration signals. The filtered resultant acceleration is then multiplied by the athlete's body mass and can be averaged over a selected time period to represent average exercise intensity (see Equation 4).

Accelerometry-derived average net force.

$$\mathrm{Av}F_{\mathrm{Net}}=BM\times \frac{\sum_{i=1}^{n}\left(\sqrt{a_{x_{i}}^{2}+a_{y_{i}}^{2}+a_{z_{i}}^{2}}\right)}{n}$$ (4)

*AvF*_*Net*_ = *Average net force, BM = body mass*, *a_xi_*, *a_yi_*, *a_zi_* = *filtered accelerations (m·s^−2^) in the mediolateral, anteroposterior, and vertical axes at sample* *i*; *n* = *number of samples*.

In addition to representing average intensity, *F*_Net_ can be accumulated over time to derive the accumulated net impulse (expressed in N·s). This involves summing the instantaneous *F*_Net_ values across the duration of activity to quantify the area under the force–time curve, providing a cumulative measure of mechanical loading that reflects total exercise volume (See Equation 5). Unlike PlayerLoad, this approach employs fully transparent computational methods and is expressed in SI units. The accumulated net impulse has been applied in basketball to quantify cumulative movement demands during gameplay (91, 92).

$$\mathrm{Accumulated}\;\mathrm{Net}\;\mathrm{Impulse}\;(N\cdot s)=\sum_{i=1}^{n}F_{\mathrm{Net},i\;}\cdot \Delta t$$ (5)

*BM = body mass (kg);* *F*_*Net*,*i*_ = *instantaneous net force derived from the product of body mass and the filtered accelerations (m·s^−2^) in the* *x*, *y*, *and* *z* *axes at sample i; n = total number of samples in the time window; Δt = 1/f_s_ = sample period (s), with f_s_ as the sampling frequency*.

These metrics benefit from transparent and scientifically justified data processing, involving dual-pass Butterworth filtering to remove gravitational acceleration and signal noise (see Section 2.1). Outcomes are expressed in SI units (N for force, N·s for impulse), ensuring interpretability and comparability. The use of standardized, SI-based, and transparent metrics like *F*_Net_ and Accel'Rate is gaining momentum in the literature (27, 91–101), reflecting a shift toward more valid and interpretable methods for quantifying human movement. These metrics support meaningful dose–response analyses and enhance cross-study and cross-athlete comparability. Broader adoption by device manufacturers could expand their practical utility; however, access currently remains limited to those capable of manually processing raw accelerometer data.

Despite these advantages, several practical limitations must also be acknowledged. *F*_Net_ assumes that the sensor's position approximates the athlete's centre of mass (COM), yet small deviations, whether due to harness movement, clothing shift, or differences in body morphology, may introduce error into the force estimate. The method also assumes constant body mass and relatively stable posture, assumptions that may be violated during activities involving substantial trunk rotation, upper-body involvement, or equipment carriage. Additionally, high-pass filtering designed to remove gravitational components can attenuate true low-frequency movement signals, and the optimal filter cut-offs may differ between sports, movement tasks, and sensor placements. Finally, deriving *F*_Net_ and accumulated impulse requires access to raw data and involves more complex processing than proprietary metrics, which may limit adoption in applied environments without dedicated analytical support.

## 4 Limitations and future research directions

This review offers a structured critique of PlayerLoad and outlines alternative approaches for quantifying accelerometry-derived external demands; however, it does not incorporate original empirical data. As such, conclusions regarding the most appropriate indicators are necessarily derived from theoretical considerations and existing literature, rather than direct validation using experimental datasets.

A further limitation is the inherent challenge of cross-device comparability. Although PlayerLoad has been highlighted as particularly sensitive to differences in filtering, sampling frequency, and sensor placement, these issues extend to virtually all accelerometry-based metrics. Any measure derived from raw acceleration data, whether based on derivative of acceleration (jerk), resultant acceleration magnitude (or any other variant), will vary depending on device hardware, data processing pipelines, and proprietary algorithms. Consequently, apparent differences between metrics may arise not from their conceptual properties but from inconsistencies in data acquisition and preprocessing. Addressing this broader issue will require coordinated efforts toward open algorithms, transparent data-processing standards, and multi-device validation studies that evaluate whether alternative metrics truly overcome the comparability and reproducibility issues that limit PlayerLoad. Future research could therefore focus on establishing cross-device and cross-context consistency alongside construct validity, ensuring that any proposed metric provides both theoretical coherence and practical interoperability.

To advance beyond these theoretical foundations, future research should prioritize empirical evaluations that clearly define the construct each metric is intended to represent (e.g., mechanical load, movement intensity, or metabolic cost) and then select appropriate criterion measures aligned with that construct. For example, mechanical validity may be assessed using force platforms, instrumented treadmills, or motion-capture–derived kinetics, whereas physiological or energetic interpretations are better evaluated using indirect calorimetry or doubly labelled water.

Building on the synthesis presented here, future studies should aim to:

1. Empirically evaluate the reliability, validity, responsiveness and measurement properties of PlayerLoad and alternative accelerometry-derived metrics across sports, movement tasks, and populations.
2. Examine the interrelationships among common athlete-monitoring indicators (e.g., accelerometry metrics, GPS variables, HR-based neuromuscular tests, perceptual responses) to determine which combinations most accurately represent external training demands.
3. Investigate the practical utility of these metrics in applied settings, including their sensitivity to fatigue, changes in mechanical demands, training adaptations, and performance outcomes.
4. Explore context- and sport-specific calibration, recognizing that the biomechanical characteristics of different sports and athlete groups may influence how accelerometry-based metrics behave and how they should be interpreted.
5. Develop integrated monitoring frameworks that combine accelerometry with complementary mechanical, physiological, and perceptual indicators to provide a more comprehensive and interpretable representation of training demands.

Together, these research priorities provide a constructive pathway for moving the field forward, bridging the gap between theoretical critique and practical implementation, and enabling more rigorous evaluation of athlete-monitoring tools used in high-performance sport.

## 5 Conclusion

Accurate measurement of human movement during exercise and sport is essential for optimizing athletic performance and informing training practices. To advance the field of sports science, it is imperative to critically evaluate commonly used yet methodologically opaque “black box” metrics. Standardizing data collection and reporting procedures will enable more meaningful comparisons across studies, teams, and individual athletes, ultimately enhancing the validity, interpretability, and impact of movement-derived metrics. Despite its widespread use, the PlayerLoad metric has several well-documented limitations that constrain its scientific and practical utility. By consolidating evidence across signal processing, biomechanics, and applied sport settings, this review offers a refined conceptual framework for evaluating the utility and limitations of PlayerLoad, thereby extending beyond descriptive summaries of previous work. This commentary is not intended as a critique of the researchers or practitioners who use PlayerLoad, but rather as a constructive contribution to the ongoing development of robust and transparent measurement tools. We encourage open-source metrics for the benefit of science, and we caution practitioners to be skeptical of proprietary metrics until they are fully understood and validated. We have outlined several alternative accelerometer-derived metrics that are grounded in stronger scientific principles and offer clearer, more interpretable outputs. By adopting such alternatives, manufacturers, researchers, coaches, and athletes can improve the accuracy and relevance of athlete monitoring practices. In doing so, they contribute to a more evidence-based and methodologically sound approach to performance assessment and enhancement.
